# Macrophage PPAR-γ suppresses long-term lung fibrotic sequelae following acute influenza infection

**DOI:** 10.1371/journal.pone.0223430

**Published:** 2019-10-04

**Authors:** Su Huang, Nick P. Goplen, Bibo Zhu, In Su Cheon, Youngmin Son, Zheng Wang, Chaofan Li, Qigang Dai, Li Jiang, Min Xiang, Eva M. Carmona, Robert Vassallo, Andrew H. Limper, Jie Sun

**Affiliations:** 1 Thoracic Diseases Research Unit, Division of Pulmonary and Critical Care Medicine, Department of Medicine, Mayo Clinic College of Medicine and Science, Rochester, Rochester, Minnesota, United States of America; 2 Department of Immunology, Mayo Clinic College of Medicine and Science, Rochester, Rochester, Minnesota, United States of America; University of Alabama at Birmingham, UNITED STATES

## Abstract

Influenza virus causes a heterogeneous respiratory infectious disease ranging from self-limiting symptoms to non-resolving pathology in the lungs. Worldwide, seasonal influenza infections claim ~500,000 lives annually. Recent reports describe pathologic pulmonary sequelae that result in remodeling the architecture of lung parenchyma following respiratory infections. These dysfunctional recovery processes that disproportionately impact the elderly have been understudied. Macrophages are involved in tissue remodeling and are critical for survival of severe influenza infection. Here, we found intrinsic deficiency of the nuclear receptor PPAR-γ in myeloid cells delayed the resolution of pulmonary inflammation following influenza infection. Mice with myeloid cell-specific PPAR-γ deficiency subsequently presented with increased influenza-induced deposition of pulmonary collagen compared to control mice. This dysfunctional lung remodeling was progressive and sustained for at least 3 months following infection of mice with myeloid PPAR-γ deficiency. These progressive changes were accompanied by a pro-fibrotic gene signature from lung macrophages and preceded by deficiencies in activation of genes involved with damage repair. Importantly similar aberrant gene expression patterns were also found in a secondary analysis of a study where macrophages were isolated from patients with fibrotic interstitial lung disease. Quite unexpectedly, mice with PPAR-γ deficient macrophages were more resistant to bleomycin-induced weight loss whereas extracellular matrix deposition was unaffected compared to controls. Therefore PPAR-γ expression in macrophages may be a pathogen-specific limiter of organ recovery rather than a ubiquitous effector pathway in response to generic damage.

## Introduction

Influenza virus infects 5–10% of adults and 20–30% of children worldwide [1]. Though seasonal influenza is often thought of as a rapidly cleared self-limiting infection, evidence is mounting that long-term sequelae can accumulate despite clearance of infectious viral particles [2–6]. A long-awaited plausible explanation for this prolonged immune stimulus may be the recent discovery of transcriptionally active viral RNA remnants. Depots of active RNA remnants are associated with pulmonary regions of chronic inflammation and lesions dispersed throughout the parenchyma and are observable months after clearance of influenza virus [2]. Prolonged activation of innate and/or adaptive immune system modulates severity and longevity of influenza-associated diseases [7–12]. Accordingly, activation of naïve and memory influenza-specific CD8 T cells can occur in lung draining lymph-nodes months after the lung is cleared of infectious virus particles [13–15]. It is therefore plausible that chronic immune stimuli may be driving abnormal remodeling and pathologies in the lung following severe influenza infection.

Respiratory viruses, including influenza, have indeed been shown to cause abnormal remodeling of lung parenchyma dependent on the severity of infection [2, 16–18]. The lungs are responsible for gas exchange; coupled with circulation, this results in supplying the body with oxygen for cellular respiration. Replacing pulmonary parenchyma (alveoli) with scar tissue therefore has functional consequences. Similar to physiological lung changes in chronic asthma models, matrix deposition, mucus secretion, and airway hypersensitivity to chronic stimuli have been demonstrated weeks after influenza clearance [2, 16, 17, 19]. Consequences of the pathologic sequelae include airway dysfunction, non-resolving inflammatory foci including tertiary lymphoid-like structures, and chronic lesions in the face of prolonged activation of the innate and adaptive immune systems. While these are now established chronic hallmarks of severe influenza infection in animal models with primate correlates [2, 16–18], how the immune system tunes infection response from benign to pathologic remodeling of the lung parenchyma, is not clear.

The innate immune system responds to damage- and pathogen-associated molecular patterns (DAMPs and PAMPs) to simultaneously initiate anti-microbial defense, immune-response resolution, and repair mechanisms [4, 20–22]. Repair of immune response induced damage occurs early after injury and is predisposed to dysfunction with age [23–26]. Macrophages are a versatile and plastic cell type capable of homeostatic, pro-inflammatory, pro-repair, and fibrotic promoting functions all in the same immune response [27–30]. Following influenza infection, the lung macrophage compartment is a heterogeneous population of monocyte-derived and embryonic derived cells with similar surface phenotypes, but distinct transcriptomes [31, 32].

PPAR-γ is a member of the nuclear hormone receptor superfamily. It is a lipid-sensing nuclear transcription factor with many intracellular ligands, including essential fatty acids and eicosanoids. PPAR-γ is known to regulate downstream gene expression mediating lipid storage and glucose homeostasis, cell growth, proliferation, and differentiation [33, 34]. Outside of adipocytes, under homoeostatic conditions, protein levels of PPAR-γ in the lung primarily derive from macrophages where it is required for their differentiation and normal recovery of influenza-infected animals [33, 35, 36]. Following influenza infection, PPAR-γ expression in alveolar macrophages is down-regulated in a manner dependent on intrinsic Type I interferon receptor signaling [37, 38]. PPAR-γ expression in AM was critical for the suppression of exaggerated antiviral and inflammatory responses of AM following influenza virus infection [37, 38]. As such, myeloid PPAR-γ deficiency resulted in enhanced acute host morbidity and delayed tissue recovery following influenza virus infection [37, 38].

Here, we demonstrate that myeloid cell PPAR-γ is important for recovery of influenza- induced injury. Whereas inflammatory and repair responses were largely resolved in wild type (WT) mice 30 days post-infection, they are sustained and delayed in myeloid PPAR-γ deficient mice. Subsequently, 60 and 90 days post-infection lungs from animals with PPAR-γ deficient macrophages exhibited a pro-fibrotic gene signature coupled with increased collagen deposition compared to wild type lungs. These findings of pro-fibrosis and pro-inflammatory signatures aligned well with macrophages from pulmonary fibrosis patients, who experience non-resolving progressive lung remodeling. However, mice with the same genetic defect exhibited enhanced recovery from bleomycin induced weight loss, with no effects on collagen deposition during the resolution phase of bleomycin-induced fibrosis. Thus, expression of PPAR-γ in macrophages limits pathogenic influenza-induced lung remodeling. Our data indicate existing agonists of PPAR-γ may alter the severity of influenza disease in the elderly that disproportionately experience long-term complications from seasonal flu infections.

## Methods

### Mouse and infection

WT C57/BL6, Lyz2-cre, *Pparg*^*fl/fl*^, were purchased from the Jackson Laboratory and bred in house. *Pparg*^*ΔLyz2*^ (PPAR-γ cKO) mice were generated by crossing *Pparg*^*fl/fl*^ (WT) mice with Lyz2-cre mice. All mice housed in a specific pathogen-free environment. For influenza virus infection, influenza A/PR8/34 strain (~200 pfu/mouse) was diluted in FBS-free DMEM media (Corning) on ice and inoculated in ketamine-anesthetized mice through intranasal route as described before [39]. All animals are sex and age matched. Animal experiments and procedures were approved by Mayo Clinic Institutional Animal Care and Use Committee (IACUC study #: A00002027 and A00002246). Animals were sacrificed with overdose of ketamine followed with cervical dislocation as approved in the IACUC protocols. Animal numbers used in each experiment are indicated in the figure legends and total animal numbers used in the experiments were 124 mice. Infected animals are monitored daily. Experimental mice were unrestricted with food or water. Moribund mice, mice under severe respiratory distress or mice lost more than 30% of weight loss were humanely sacrificed according to the IACUC protocols listed.

### Bleomycin treatment

*Pparg*^*fl/fl*^ or *Pparg*^*ΔLyz2*^ mice were used at 12 weeks old. All mice were anesthetized with ketamine before bleomycin administration. Bleomycin was dissolved in saline and was given at the dosage of ~2.4 units per kilogram body weight (0.072 unit/mouse in 50 μl saline). Bleomycin was given by intratracheal instillation through an aerosolizer under visualization for each mouse [40]. After bleomycin administration, antisedan (atipamezole hydrochloride) was immediately given by IP (100μl diluted in 5 ml saline, 200μl for each mouse) and mice were put on heat pad with oxygen supply. Body weight was monitored in following days. At day 42 post bleomycin treatment, lungs were removed for determination of hydroxyproline levels in the whole lung.

### Lung histopathology

Following euthanasia, mice were perfused with PBS (10 mL) via the right ventricle. 10% paraformaldehyde (PF) was then gently instilled into the lung and left inflated for 1 minute before excising and moving lobe to 10% PF for 48 hours followed by transfer to ethanol (70%). Samples were shipped to Mayo Clinic Histology Core Lab (Scottsdale, AZ) where they were embedded in paraffin and 5 um sections were cut for Hematoxylin and eosin (H&E) stain.

### Quantitative RT-PCR

mRNA from cultured AM (pooled from multiple mice), *in vivo* sorted AM (pooled from multiple mice) or homogenates from individual lungs as indicated in the text was isolated with Total RNA purification kit (Sigma) and treated with DNase I (Invitrogen) as described [41]. Random primers (Invitrogen) and MMLV reverse transcriptase (Invitrogen) were used to synthesize first-strand cDNAs from equivalent amounts of RNA from each sample. RT-PCR was performed with Fast SYBR Green PCR Master Mix (Applied Biosystems). qPCR was conducted in duplicates in QuantStudio3 (Applied Bioscience). Data were generated with the comparative threshold cycle (Delta CT) method by normalizing to hypoxanthine phosphoribosyltransferase (HPRT). Sequences of primers will be provided if requested.

### RT^2^ profiler PCR array

Total RNA from lung tissue or AM was extracted as described above. Equal amount of total RNA was used for the synthesis of first strand cDNA with kit from Qiagen. First strand cDNA was mixed with 2xFast SYBR Green Master Mix (Applied Bioscience) and water in a formula directed in the manual. 25 μl of the mixture was added into each well of the 96 well plate provided by manufacture. The wells in the plate include different primers in each well to detect 84 target genes, housekeeping genes, negative and positive control genes. qPCR was conducted in QuantStudio3 (Applied Bioscience). Obtained raw data was analyzed in software provided by Qiagen (accessible online on the website of Qiagen). Following the instruction step by step, upload Excel file, designating control group, select housekeeping gene to normalize result and calculate the relative expression quantity.

### Hydroxyproline assay

Hydroxyproline assay for determining total lung collagen content was performed as previously reported [6]. Briefly, isolated whole lungs were cut into small pieces. Lung tissue was hydrolyzed in 1 ml 6 M HCL. The hydro lysate was cooled down to room temperature. Hydroxyproline standard solution was purchased from Sigma. Diluted standard solution were added into different wells in the assay plate for generation of standard curve. Freshly-made Chloramine-T Solution (2.0 ml N-Propanol, 0.282g Chloramine-T, 2.0ml H_2_O in 20ml citrate acetate buffer) was added into each well and the mixture was incubated at room temperature for 20 minutes. Then, 100 μl of fresh Ehrlich solution (4.5g 4-dimethylaminobenzaldehyde in 18.6 ml N-Propanol and 7.8 ml perchloric acid) was added into each well. Following that, the assay plate was placed at a 60°C oven for 60 minutes before reading at 560 nm wavelength in Thermax plate reader.

### Flow cytometry and flow sorting

Fluorescence-conjugated FACS Abs were purchased from Biolegend or Tonbo Bioscience. Lung single cell suspensions were prepared according to previous publications [42, 43]. Lung cells were then washed with FACS buffer (PBS, 2 mM EDTA, 2% FBS, 0.09% Sodium Azide) and stained with CD64, Ly6G, CD11b, Siglec F, CD11c and MertK. Samples were collected on FACS Attune NXT flow cytometer (Life technologies) and analyzed using Flow Jo software (Tree Star). Total lung macrophages were defined as CD64^+^ Ly6G^-^. For cell sorting, total lung cells were pooled from infected WT or PPAR-γ cKO mice and stained with CD45, Ly6G, CD64 and MertK. CD45^+^ Ly6G^-^ CD64^+^ MertK^+^ lung total macrophages (including resident macrophages and recruited macrophages) were sorted by BD FACSAria II. mRNA was extracted from sorted macrophages and realtime RT-PCR was performed as above.

### Statistical analysis

Data are mean ± SEM of values from individual mice (*in vivo* experiments). Unpaired two-tailed Student’s t-test (two group comparison), Mann-Whitney test, One-way ANOVA with Tukey multiple comparison test (multi-group comparison) and Multiple t-tests (weight loss) were used to determine statistical significance by GraphPad Prism software. We consider *P* values < 0.05 as significant.

### Data availability

The dataset we used has been publicly deposited in the GEO database: GSE49072 by Shi et al [44].

## Results

### Myeloid PPAR-γ deficiency leads to enhanced lung inflammation and collagen deposition following the resolution of influenza virus infection

**Fig 1:**
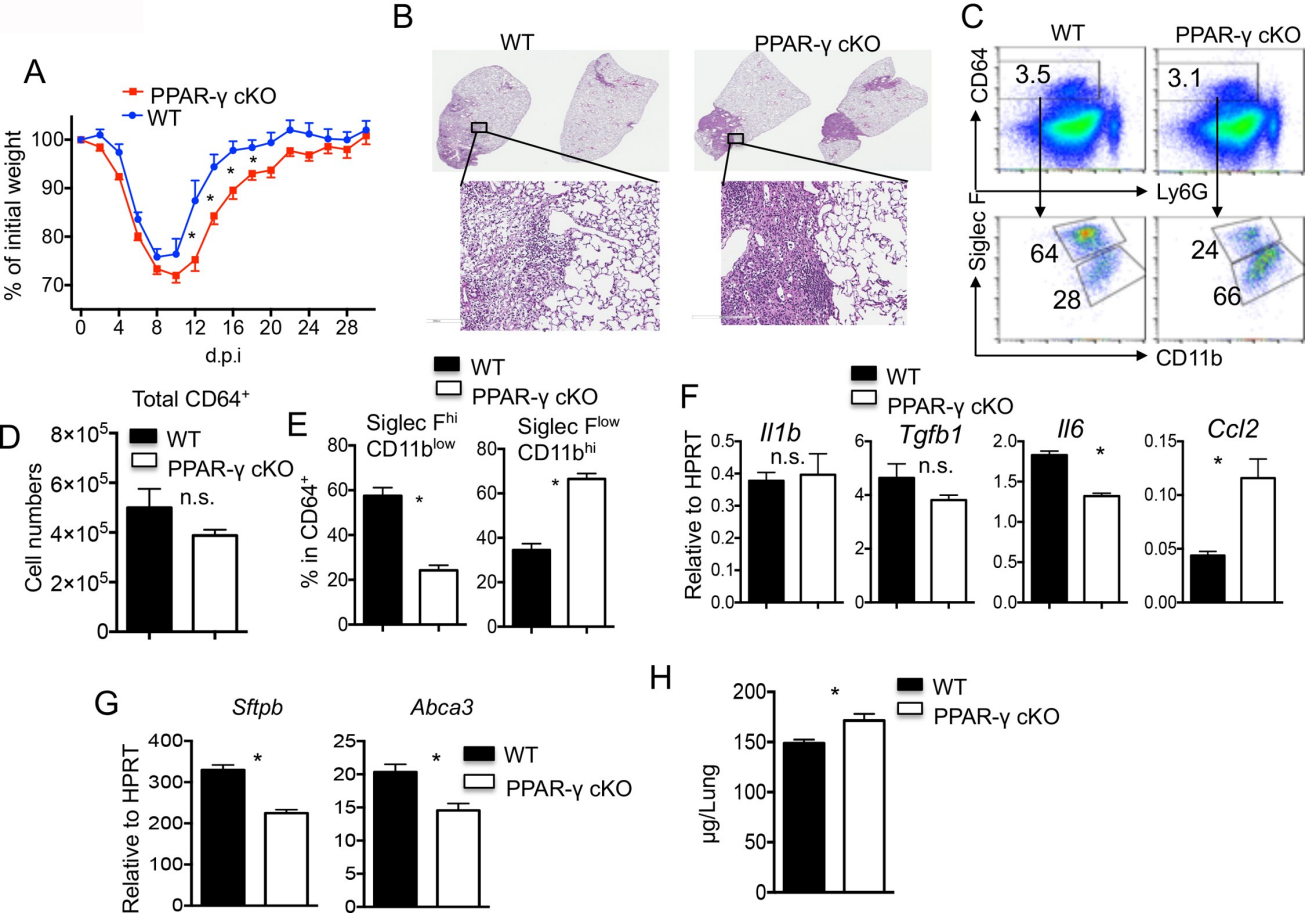
Myeloid PPAR-γ deficiency causes impaired lung inflammation resolution and enhanced collagen deposition. WT (*Pparg*^*fl/fl*^) or PPAR-γ cKO (*Pparg*^*ΔLyz2*^) mice were infected with influenza virus. Mice were sacrificed for various analysis at 30 d.p.i. (**A**) Host morbidity (% of initial weight) was monitored every other day till 30 d.p.i. (**B**) H.E stainning of lung sections collected from WT or PPAR-γ cKO mice. Low magnitude of whole left lung section image and high magnitude of indicated section images are depicted. (**C**) Lung cells from WT or PPAR-γ cKO mice were stained with CD64, Ly6G, Siglec F and CD11b and analyzed by flow cytometry. CD64^+^ macrophages were then subdivided into Siglec F^hi^ CD11b^low^ and Siglec F^low^ CD11b^hi^ populations. (**D**) Total numbers of CD64^+^ macrophages in the lungs from WT or PPAR-γ cKO mice were enumerated by flow cytometry. (**E**) Percentages of Siglec F^hi^ CD11b^low^ or Siglec F^low^ CD11b^hi^ macrophage populations were determined by flow cytometry. (**F**) *Il1b*, *Tgfb1*, *Il6* and *Ccl2* gene expression levels in the lungs from WT or PPAR-γ cKO mice were determined by realtime RT-PCR. (**G**) ATII gene, *Sftpb* and *Abca3*, expression levels in the lungs from WT or PPAR-γ cKO mice were determined by realtime RT-PCR. (**H**) Collagen content in the lungs from WT or PPAR-γ cKO mice was measured by Hydroxyproline assay. Data are representative of at least two independent experiments (n = 2–4 mice/group/experiment). Statistical differences are indicated. *, *P* < 0.05 (two tailed t-test).

We previously demonstrated that the transcription factor PPAR-γ was downregulated in alveolar macrophages (AM) following influenza virus infection [37, 38]. Within the lung compartment, myeloid cells, particularly AM expressed high levels of PPAR-γ (S1 Fig). Strikingly, myeloid deficiency of PPAR-γ (*Pparg*^*ΔLyz*^) resulted in increased host disease development, enhanced pulmonary inflammation and impaired tissue recovery at 15 days post influenza virus infection (d.p.i.) [37, 38]. Furthermore, *Pparg*^*ΔLyz*^ mice showed delayed host weight recovery. However, majority of the infected *Pparg*^*ΔLyz*^ survived and recovered most of their weight loss at 15 d.p.i. [37, 38]. Interestingly, recent reports have suggested that acute influenza infection can lead to chronic development of pathology, including fibrotic responses in the lungs [2–6]. We wondered whether myeloid deficiency of PPAR-γ leads to exacerbated development of chronic lung sequela following acute influenza infection. To this end, we infected WT (*Pparg*^*fl/fl*^) and *Pparg*^*ΔLyz*^ mice with sublethal influenza virus A PR/8 strain. Consistent with our previous observations, *Pparg*^*ΔLyz*^ mice displayed increased morbidity and delayed recovery compared to control mice (Fig 1A) [37, 38]. By 30 d.p.i., however, *Pparg*^*ΔLyz*^ mice exhibited comparable weight gain as WT mice (Fig 1A). To examine whether myeloid PPAR-γ deficiency causes long-term effects on lung tissue following influenza infection, we examined the lung tissue secions at day 30 p.i. H&E staining of lung tissue sections revealed lesions in lungs from PPAR-γ cKO mice were far denser than controls indicating enhanced non-resolving inflammation and tissue injury (Fig 1B). Since PPAR-γ mainly expressed in macrophages but not neutrophils, we next examined macrophage composition in the lungs from WT or PPAR-γ cKO mice at 30 d.p.i. We found that total lung macrophage numbers (CD64^+^) were comparable between WT and PPAR-γ cKO mice (Fig 1C and 1D). However, WT mice had enhanced percentages of Siglec F^hi^ CD11b^low^ macrophages, representative of tissue-resident alveolar macrophages, while PPAR-γ cKO mice showed higher percentages of Siglec F^low^ CD11b^hi^ macrophages, a phenotype that associates with recruited monocyte-derived macrophages [32]. We next examined that cytokine gene expression in the lungs and found that lungs from WT and PPAR-γ cKO mice show comparable *Il1b* and *Tgfb1* expression, but PPAR-γ cKO mice have modestly diminished *Il6* expression and enhanced *Ccl2* expression, suggesting that myeloid deficiency of PPAR-γ altered inflammatory responses in the lungs following the clearance of influenza virus infection (Fig 1F). Strikingly, at this time-point, late deficits in repair in the lung were observed by a decrease in *Sftpb* and *Abca3;* genes associated with type II airway epithelial cells (ATII), which are present in increased numbers in repaired tissue following injury (Fig 1G). Since persistent injury often drives tissue fibrotic responses, we next examined lung collagen content as the surrogate of lung fibrosis. To this end, hydroxyproline content, which was frequently used as a surrogate for collagen deposition, in the whole lungs of WT or PPAR-γ cKO mice was quantified at 30 d.p.i. We found that collagen deposition was quantitatively increased in whole lungs from PPAR-γ cKO mice compared to those of WT mice. (Fig 1H). Together, these data indicate that myeloid PPAR-γ deficiency results in delayed host weight recovery, altered macrophage and inflammatory responses and impaired repair of the lungs, which ultimately result in enhanced collagen deposition weeks following initial influenza exposure.

### PPAR-γ deficient macrophages exhibited pro-inflammatory and pro-fibrotic gene activation

**Fig 2:**
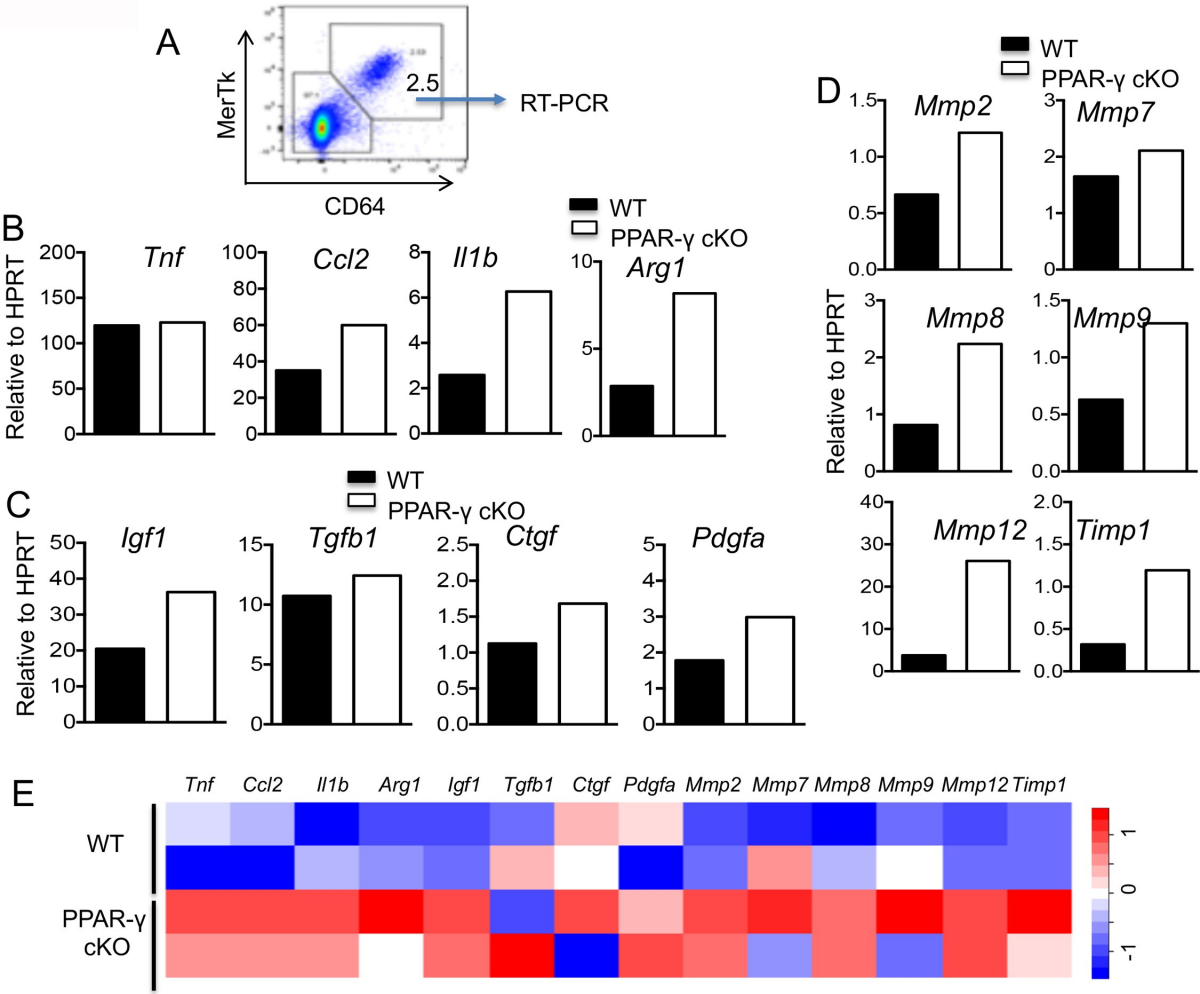
PPAR-γ deficient macrophages exhibited pro-inflammatory and pro-fibrotic gene expression. WT (*Pparg*^*fl/fl*^) or PPAR-γ cKO (*Pparg*^*ΔLyz2*^) mice were infected with influenza. Mice were sacrificed for macrophage isolation at 30 d.p.i. (**A**) Lung cells were stained with anti-CD45, anti-Ly6G, anti-MerTK and anti-CD64. MerTK^+^/CD64^+^ lung macrophages in CD45^+^ Ly6G^-^ cells were sorted by flow cytometry for realtime RT-PCR analysis of gene expression. (**B**) Expression of *Tnf*, *Ccl2*, *Il1b* and *Arg1* in lung macrophages (isolated from pooled 3–4 mice/group/experiment) by realtime RT-PCR. (**C**) Expression of growth factors *Igf1*, *Pdgfa*, *Tgfb* and *Ctgf* in sorted lung macrophages (isolated from pooled 3–4 mice/group/experiment) by realtime RT-PCR. (**D**) Expression of several tissue remodeling enzymes and fibrotic genes *Mmp2*, *Mmp8*, *Mmp12*, *Mmp7*, *Mmp9* and *Timp1* in sorted lung macrophages (isolated from pooled 3–4 mice/group/experiment) by realtime RT-PCR. (**E**) Heat-map expression of the pro-inflammatory and profibrotic genes shown in B-D in lung alveolar macrophages from adult WT or conditional PPAR-γ-deficient mice (*Pparg*^*ΔCD11c*^) in published microarray dataset (GSE60249). Data are representative of two independent experiments of pooled lung macrophages from 3–4 mice except (**E**).

To assess the role of PPAR-γ deficient macrophages in the aberrant recovery following influenza virus infection, we sorted pulmonary macrophages (CD45^+^/ly6G^-^/CD64^+^/MerTK^+^) from WT and myeloid PPAR-γ deficient mice at day 30 p.i. (isolated from pooled 3–4 mice/group/experiment) (Fig 2A), and examined the expression of several genes associated with inflammation and fibrosis. Expression of *Il1b*, *Ccl2* and *Arg1* but not *Tnf* was elevated in PPAR-γ cKO macrophages (Fig 2B). Furthermore, we observed PPAR-γ deficient macrophages exhibited moderate increased expression of epithelial and fibroblast growth factors (*Igf1*, *Pdgfa and Ctgf*) (Fig 2C) and enhanced lung remodeling factors including *Mmp2*, *Mmp7*, *Mmp8*, *Mmp9*, *Mmp12 and Timp1* (Fig 2D). In accordance, analysis of published microarray dataset (GSE60249 [36]) has revealed that PPAR-γ deficiency leaded to the upregulation of these pro-inflammatory and/or pro-fibrotic genes in AM even in the absence of influenza infection (Fig 2E). Thus, loss of PPAR-γ in macrophages led to sustained macrophage-intrinsic inflammation and lung remodeling programs at a time when they are largely resolved or tempered in wild type control macrophages.

### Macrophages from human pulmonary fibrosis patients have overlapping repair, pro-inflammatory, and fibrotic gene signatures with PPAR-γ deficient macrophages

**Fig 3:**
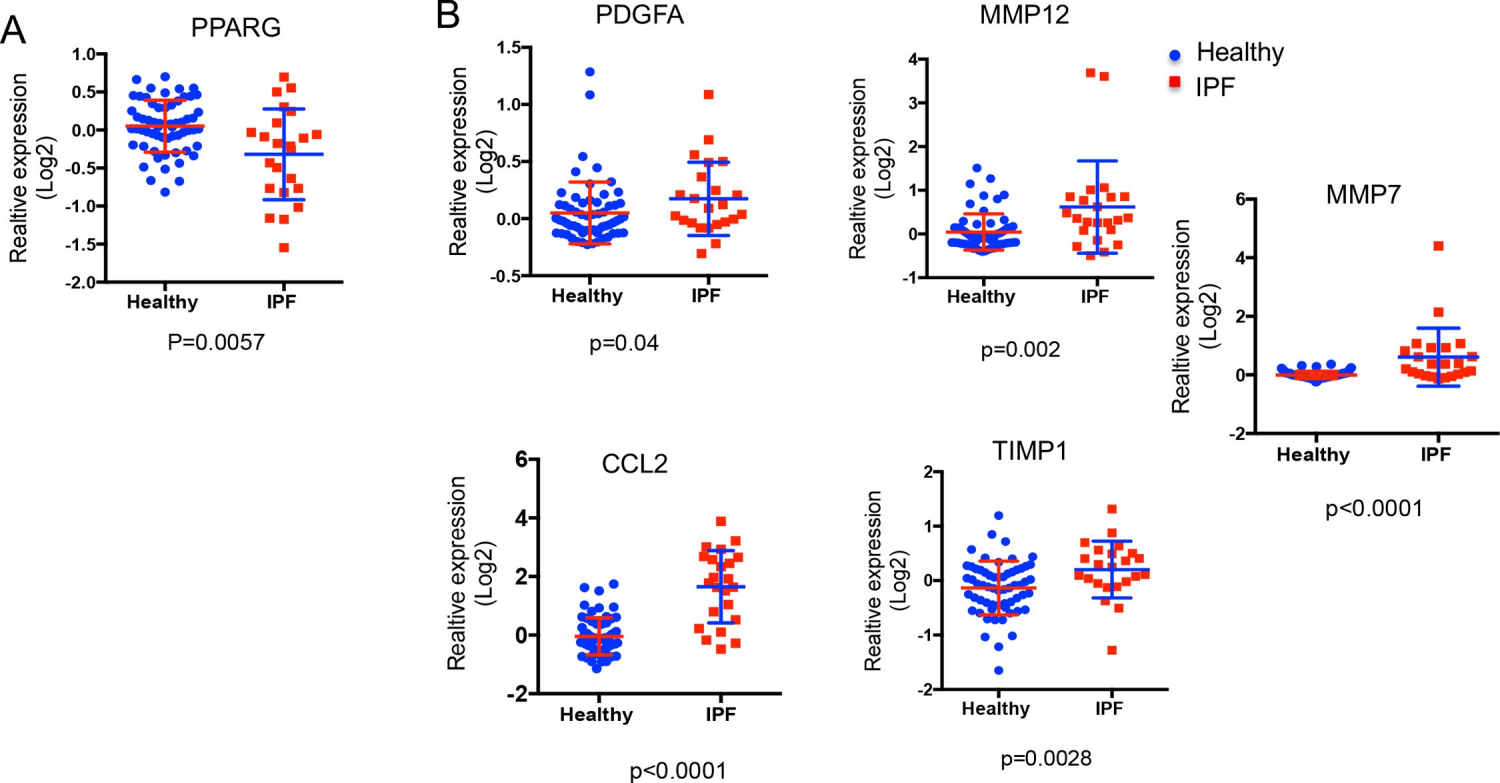
Macrophages from human pulmonary fibrosis patients exhibited diminished PPARG and dysregulated pro-inflammatory and pro-fibrotic gene expression. PPARG, pro-inflammatory and pro-fibrotic gene expression in lung macrophages isolated from 61 healthy volunteers or 23 pulmonary fibrosis patients in the GSE49072 microarray datasets. (**A**) PPARG expression in lung alveolar macrophages from healthy or pulmonary fibrosis patients. (**B**) MMP7, MMP12, TIMP1, CCL2 or PDGFA expression in lung alveolar macrophages from healthy or pulmonary fibrosis patients. Statistical values are indicated (two-tailed Mann-Whitney test).

Idiopathic pulmonary fibrosis (IPF) is a chronic, progressive, fibrotic lung disease of unknown etiology. The roles of macrophages in IPF development and/or pathogenesis have been increasingly appreciated [5, 30]. However, the underlying molecular mechanisms regulating macrophage function in IPF remain largely elusive. Since we observed macrophage PPAR-γ deficiency increased lung collagen deposition, we wondered whether IPF patients exhibited altered PPAR-γ expression in macrophages. A secondary analysis from a gene-array expression profile database of macrophages sorted from healthy controls and pulmonary fibrosis patients indicates decreased PPAR-γ expression in patient macrophages (GEO #: GSE49072, 61 healthy volunteers and 23 pulmonary fibrosis patients) (Fig 3A). These data suggest that dysregulated PPAR-γ expression in pulmonary macrophages may play a role in lung fibrosis development and/or pathogenesis. In consistent with the idea, as was indicated in PPAR-γ deficient macrophages from influenza infected mice 30 days after viral infection (20 days after viral clearance), there were differences in pro-inflammatory (*Ccl2*), growth factor (*Pdgfa*), and fibrosis-associated gene expression (*Mmp7*, *Mmp12*, and *Timp1*) in macrophages from human IPF patients versus healthy control (Fig 3B). The parallel changes of PPAR-γ and a group of fibrosis related genes observed between murine PPAR-γ deficient macrophages (from influenza virus infection) and macrophages from pulmonary fibrosis patients might reflect important roles of PPAR-γ in macrophages in regulating the development and/or maintenance of progressive fibrosis leading to respiratory failure in IPF patients.

### Myeloid PPAR-γ deficiency facilitates chronic influenza-induced pulmonary fibrosis

**Fig 4:**
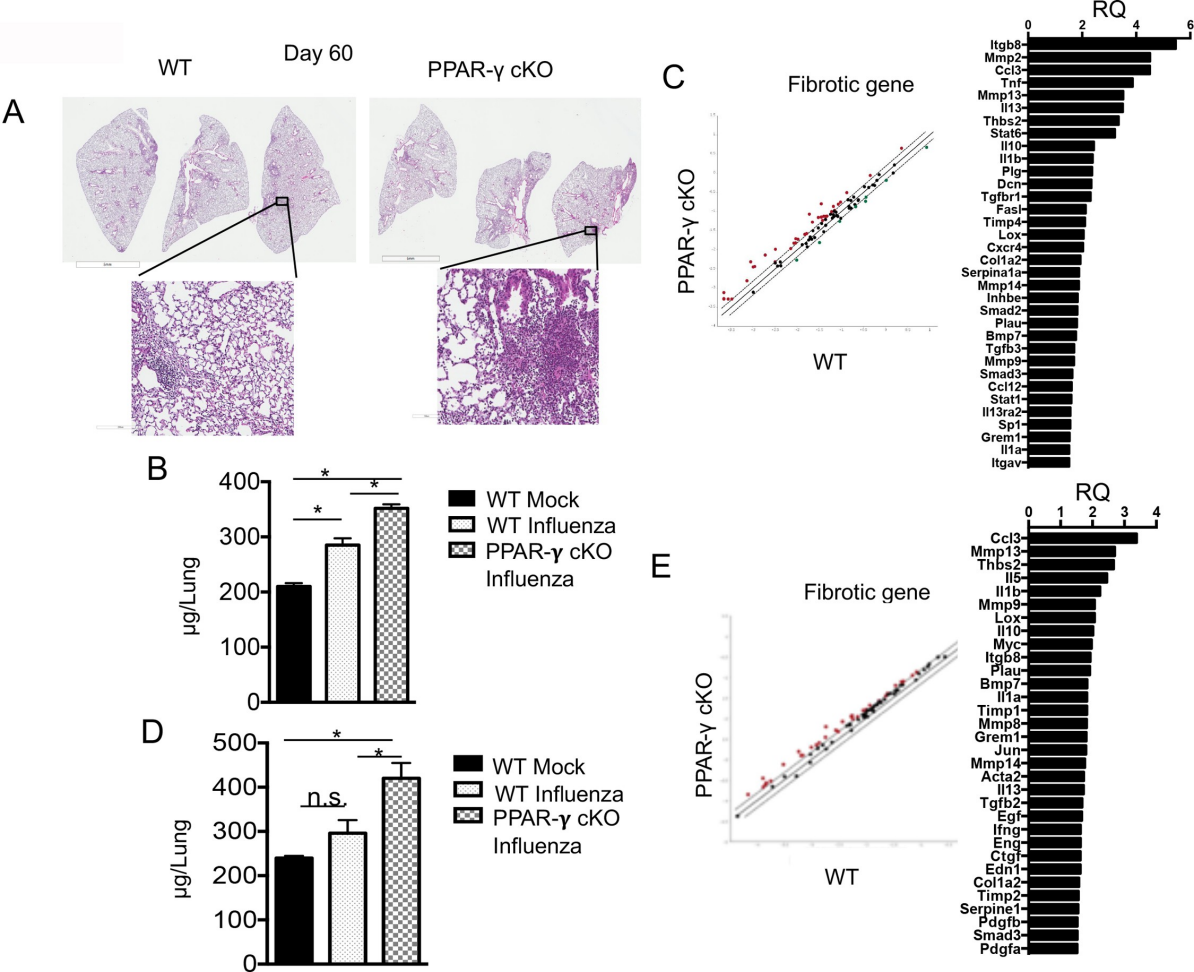
Myeloid PPAR-γ deficiency leads to unresolved persistent fibrosis. (**A**) WT (*Pparg*^*fl/fl*^) or PPAR-γ cKO (*Pparg*^*ΔLyz2*^) mice were infected with influenza virus. H&E staining of lung sections from WT or PPAR-γ cKO mice at day 60 p.i. (**B**) WT (*Pparg*^*fl/fl*^) or PPAR-γ cKO (*Pparg*^*ΔLyz2*^) mice were treated with saline (mock) or infected with influenza as indicated. Collagen content in the whole lungs from indicated groups mice was measured by Hydroxyproline assay at day 60 p.i. (**C**) WT (*Pparg*^*fl/fl*^) or PPAR-γ cKO (*Pparg*^*ΔLyz2*^) mice were infected with influenza virus. Lung fibrotic genes expression was measured by QIAGEN Fibrosis RT^2^ RT-PCR array at day 60 p.i. Red dots, genes upregulated in PPAR-γ cKO lungs, green dots, genes downregulated in PPAR-γ cKO lungs. (**D**) WT (*Pparg*^*fl/fl*^) or PPAR-γ cKO (*Pparg*^*ΔLyz2*^) mice were treated with saline (mock) or infected with influenza virus as indicated. Collagen content in the lungs from indicated groups mice was measured by Hydroxyproline assay at day 90 p.i. (**E**) WT (*Pparg*^*fl/fl*^) or PPAR-γ cKO (*Pparg*^*ΔLyz2*^) mice were infected with influenza virus. Lung fibrotic genes expression was measured by QIAGEN Fibrosis RT^2^ RT-PCR array at day 90 p.i. Red dots, genes upregulated in PPAR-γ cKO lungs, green dots, genes downregulated in PPAR-γ cKO lungs. Data are representative of two independent experiments (3–5 mice/group/experiment). Statistical differences are indicated. *, *P* < 0.05 (one-way ANOVA).

To further assess the effect of myeloid deficiency of PPAR-γ on recovery following influenza infection, we examined lesions and collagen deposition in the lung tissue at 60 days p.i. H&E staining of lung tissue sections revealed sustained inflammation and damage from mice with PPAR-γ deficient macrophages compared to those of WT lungs that were largely resolved in these regards at 60 days p.i. (Fig 4A). At this time, higher collagen deposition was found in lungs of mice with myeloid PPAR-γ deficiency compared to those of WT mice (Fig 4B). Consistent with this observation, RT-qPCR array data exhibited globally enhanced pro-inflammatory and pro-fibrotic gene profiles in lungs of myeloid PPAR-γ deficient mice (Fig 4C), confirming that macrophage PPAR-γ has functional consequences for the lung at the level of gene transcription. To see if this persisted another month, we measured collagen content and fibrotic gene transcription in the lungs of WT or PPAR-γ cKO mice at 90 d.p.i. (Fig 4D and 4E). Notably, influenza-infected control animals had a measurable increase in total lung collagen at 60 d.p.i. relative to non-infected counterparts, but this is largely resolved by day 90 p.i., suggesting that lung fibrotic responses due to influenza infection were largely resolved in WT mice at three months after infection. Remarkably, lungs from PPAR-γ cKO animals exhibited enhanced lung collagen content than the lungs from non-infected or influenza-infected WT mice, suggesting that myeloid PPAR-γ deficiency exacerbated extracellular matrix deposition and/or delayed the clearance of deposited collagen. The data likely indicate myeloid PPAR-γ deficiency leads to persistent progressive pro-fibrotic activity in the lungs following influenza infection.

### Mice with PPAR-γ deficient macrophages are more resistant to bleomycin-induced weight loss

**Fig 5:**
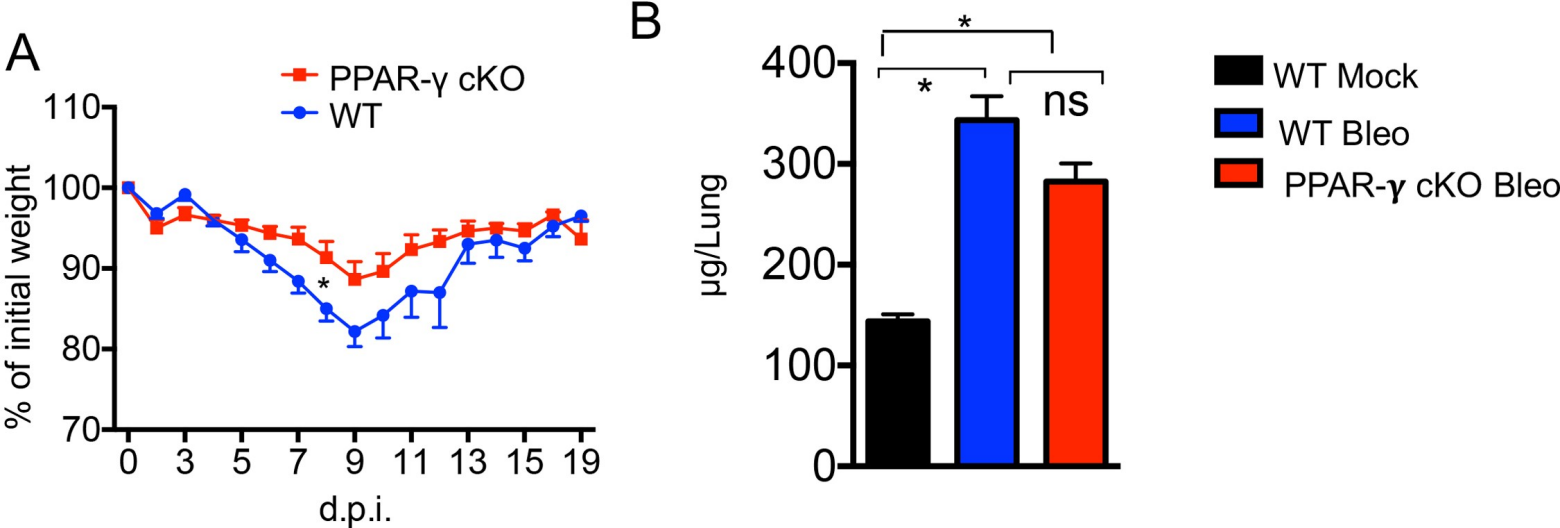
Mice with myeloid PPAR-γ deficiency were more resistant to bleomycin damage. WT (*Pparg*^*fl/fl*^) or PPAR-γ cKO (*Pparg*^*ΔLyz2*^) mice were inoculated with bleomycin (0.072 Unit/mouse). (**A**) Host morbidity (% of initial weight loss) was monitored following bleomycin treatment. (**B**) Collagen content in whole lungs from WT or PPAR-γ cKO mice was measured by Hydroxyproline assay at 42 days after bleomycin treatment. Data are representative of two independent experiments (3–5 mice/group/experiment). Statistical differences are indicated. *, *P* < 0.05. ns, not significant. (A. t test or B. one-way ANOVA).

To examine if the PPAR-γ signaling pathway in macrophages is involved in other damage induced pro-fibrotic responses, we compared the responses induced by intratracheal instillation of bleomycin between wild type and PPAR-γ cKO mice. In contrast to the enhanced weight loss observed in PPAR-γ cKO mice following influenza virus infection, we found that myeloid PPAR-γ deficiency resulted in decreased host morbidity following bleomycin inoculation (Fig 5A). These data suggest that macrophage PPAR-γ may differentially regulate the acute lung inflammation and injury following influenza or bleomycin treatment (Fig 5A). Previous reports have demonstrated that peak of lung fibrosis development following bleomycin treatment is around 2–4 weeks [45, 46]. To study the roles of myeloid PPAR-γ deficiency in regulating the resolution of pulmonary fibrosis, we compared total lung collagen content in the lungs between WT and PPAR-γ cKO mice at 6 weeks post bleomycin inoculation (Fig 5B). We found that myeloid PPAR-γ deficiency did not resulted in increased collagen deposition following bleomycin treatment, which is in sharp contrast to what we have observed in the influenza infection model. Thus, macrophage PPAR-γ may not be an effector pathway for all types of injury and may be limited to microbial responses in the lung.

## Discussion

Emerging evidence has suggested that acute respiratory viral infections can cause chronic disease sequela including fibrotic responses months following the clearance of infectious viruses [2–6]. Likewise, post influenza fibrotic responses following the resolution of acute virus-induced diseases have been documented in clinical cases [47–49]. Furthermore, respiratory viral infections have been associated with the acute exacerbation of pulmonary fibrosis [50–52]. Thus, it is of significant clinical relevance to investigate the cellular and molecular mechanisms regulating the development of pulmonary fibrosis following influenza infection. In this report, we have discovered that myeloid deficiency of PPAR-γ resulted in dysregulated macrophage pro-inflammatory and pro-fibrotic responses following influenza virus infection. Strikingly, we observed that persistent fibrotic responses were persistent in myeloid PPAR-γ deficient mice for at least 3 months following infection. Previously, we have shown that macrophage PPAR-γ is a critical inhibitor of acute disease development (before day 15) following influenza virus infection [37, 38]. Together, these data suggest that macrophage PPAR-γ is critical for the suppression of both acute and chronic pathogenesis following primary influenza virus infection.

The roles of macrophage in regulating the development of tissue fibrosis have been well-established. Particularly, alternative-activated macrophages (M2) have been demonstrated to promote fibrosis development in multi-organs including the lungs [5, 30]. PPAR-γ was shown before as a critical regulator for M2 differentiation following IL-4 treatment [33]. However, PPAR-γ deficiency did not alter the expression of M2 genes, *Arg1* and *Retnla* (encodes Relmα) expression following influenza exposure [38]. Likely, PPAR-γ deficiency increased *Arg1* expression in lung macrophages at 30 days post influenza infection. Thus, it is likely that macrophage PPAR-γ expression suppresses pulmonary fibrosis independent of M1 or M2 polarization. Indeed, emerging evidence has suggested that a dichotomous stratification of macrophage phenotypes (M1 versus M2) oversimplifies the functional heterogeneity of these highly plastic cells [5, 53, 54].

Besides the roles of macrophage polarization in fibrosis development, recent data have also suggested important roles of recruited monocyte-derived macrophages in fibrosis development [32]. Monocyte recruitment in the lung during influenza infection is largely dependent on the CCL2-CCR2 axis [55, 56]. Indeed, PPAR-γ deficient macrophages exhibited enhanced CCL2 expression and lungs from PPAR-γ cKO mice also showed increased CCL2 expression. Consistently, we observed disproportionate distribution of macrophages with resident or recruited macrophage phenotypes in PPAR-γ cKO mice. Thus, it is possible that enhanced responses of monocyte-derived macrophages may contribute to the development and/or failed resolution of pulmonary fibrosis following influenza infection. Notably, we measured pro-inflammatory and pro-fibrotic genes in total lung macrophages rather than in recruited or resident macrophage populations. We reasoned that macrophages from patient samples would contains both resident and recruited macrophage populations. Therefore, in order to compare the gene expression of the “global” macrophage population between mouse and human, we chose to examine pro-inflammatory and pro-fibrotic gene expression in the total lung macrophage population. Further studies are warranted to determine whether the increased expression of those genes was largely due to the recruitment of monocyte-derived macrophages or not. Of note, we have found that PPAR-γ deficiency in purified resident macrophages leaded to enhanced *Il1b* and *Ccl2* expression [38], suggesting that enhanced inflammatory and fibrotic genes may also occur in resident macrophage population in the absence of PPAR-γ. Nevertheless, comparison of the gene expression of the “total” macrophage compartment revealed that the effects of PPAR-γ in regulating pro-fibrotic macrophage responses may be conserved in mouse and human. Notably, Lyz2-driven Cre activities were also observed in ATII cells and PPAR-γ expression was observed in airway epithelial cells [57, 58]. Thus, it is possible that PPAR-γ expression in ATII cell may also contribute to the fibrotic phenotypes observed in myeloid PPAR-γ deficient mice following influenza virus infection. Future studies using ATII-specific deficiency of PPAR-γ is required to dissect the roles of PPAR-γ expression in ATII cells in modulating pulmonary fibrosis development following influenza virus infection.

Interestingly, the current study is one of multiple reports that has now identified a beneficial role for PPAR-γ in macrophages following influenza virus infection of mice [37,38]. This indicates that endogenous PPAR-γ agonists whose presence in the lung may temper the severity of disease sequelae. This study therefore warrants investigating the potential PPAR-γ ligands in the lung and how these differ regarding severity of non-resolving pathologies. Discovering the nature of these functional agonists in the context of influenza infection could be important for treatment of those who are highly prone to severe infection and often succumb to the resulting sequela, such as the elderly [59]. Notably, although PPAR-γ action appear to be beneficial to the hosts during primary influenza virus infection, a recent report has suggested that stimulation of PPAR-γ action could suppress efficient host anti-bacterial responses in a influenza and bacterial super infection model [60], although the macrophage-specific PPAR-γ action was not determined in this study. Nevertheless, the proper balance of PPAR-γ activities would be a key to promote efficient recovery from primary influenza infection while minimizing the risk for enhanced secondary bacterial infection.

Bleomycin is a small-molecule used to experimentally induce severe, but reversible fibrosis of the lung parenchyma. Respiratory epithelial cells are susceptible to bleomycin resulting in apoptosis following damage of genomic DNA and ensuing inflammation [61, 62]. After bleomycin insult, PPAR-γ cKO mice exhibited decreased acute weight loss. Furthermore, PPAR-γ cKO mice showed less, but no significant, collagen deposition compared with wild type mice. This is surprising as PPAR-γ ligands have been demonstrated to exhibit anti-fibrotic effects in bleomycin treated mice [63]. Currently, the difference for the fibrotic responses between the disparate models used here is not clear, but it is likely that the PPAR-γ signaling axis in pulmonary macrophages may be important for pathogen induced damage and not general damage. Further comparison on the phenotypes of PPAR-γ-deficient lung macrophages from influenza-infected mice versus those from bleomycin-treated mice is needed to confirm this idea. Nonetheless, our data imply that the early and ongoing inflammatory milieus resulting from the type of various danger signals may dictate which repair pathway(s) are used and when. Of note, we focused on our experiments at the resolution phase (i.e. 6 weeks post bleomycin treatment). Previous reports have demonstrated that peak of lung fibrosis development following bleomycin treatment is around 2–4 weeks [45, 46]. It thus remains possible that myeloid PPAR-γ deficiency may increase lung collagen deposition at the peak of pulmonary fibrosis. However, given that myeloid PPAR-γ deficiency diminished rather than increased host weight loss, we think that this possibility is low. Nonetheless, since we found overlapping patterns in dysfunctional macrophages from influenza-infected animals exhibiting fibrosis and those from IPF patients, this indicates the fibrotic sequelae found in the PPAR-γ cKO mice following acute influenza virus exposure may be a relevant model for human pulmonary fibrosis development. Given the current urgent need for better pre-clinical models for human pulmonary fibrotic diseases, the discoveries in this manuscript is of significant importance to pulmonary research.

## Supporting information

S1 Fig *Pparg* is highly expressed in alveolar macrophages compared to epithelial cells. (TIF)
