# Supplementary figures and images for: Macrophage PPAR-γ suppresses long-term lung fibrotic sequelae following acute influenza infection
Source: PLoS One. 2019 Oct 4;14(10):e0223430. doi: 10.1371/journal.pone.0223430 (PMC6777801; doi:10.1371/journal.pone.0223430)

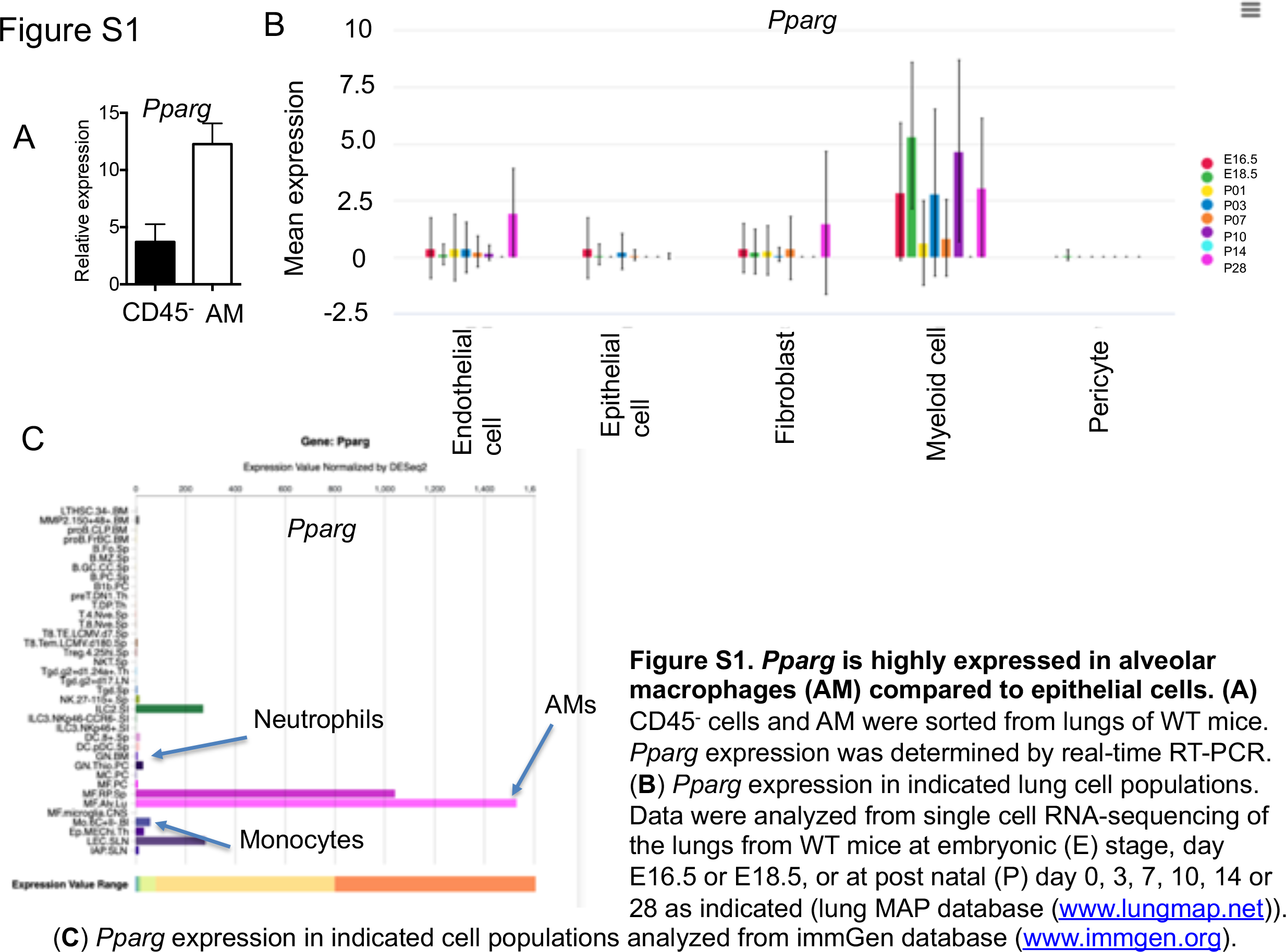

Supplement: S1 Fig — (TIF) [file pone.0223430.s001.tif]
